# Editorial: Datasets for Brain-Computer Interface Applications

**DOI:** 10.3389/fnins.2021.732165

**Published:** 2021-09-29

**Authors:** Ian Daly, Ana Matran-Fernandez, Davide Valeriani, Mikhail Lebedev, Andrea Kübler

**Affiliations:** ^1^Brain-Computer Interfacing and Neural Engineering Laboratory, School of Computer Science and Electronic Engineering, University of Essex, Colchester, United Kingdom; ^2^Neurable Inc., Boston, MA, United States; ^3^V. Zelman Center for Neurobiology and Brain Restoration, Skolkovo Institute of Science and Technology, Moscow, Russia; ^4^Department of Information and Internet Technologies of Digital Health Institute, I.M. Sechenov First Moscow State Medical University, Moscow, Russia; ^5^Institute of Psychology, Julius Maximilian University of Würzburg, Würzburg, Germany

**Keywords:** brain computer interface, datasets, EEG, functional near infrared spectroscopy, electromyogram, ERP, steady-state visual evoked potential, affect (emotion)

Non-invasive Brain-computer interfaces are an exciting new technology that provide a channel for communication between the brain and a computer system. They can be used as communication devices (Chaudhary et al., 2016; Brumberg et al., 2018), rehabilitation systems (Cervera et al., 2018), entertainment devices (Gürkök et al., 2017), and for a wide range of other applications (Finke et al., 2009; Makeig et al., 2011).

Research in non-invasive BCIs is developing rapidly and is a highly multidisciplinary field, involving, among others, neuroscientists, engineers, psychologists, computer scientists, and clinicians. Continuing development of BCI technology relies on advances made in each of these fields, which individually and collectively can contribute to improving all aspects of BCI systems including signal acquisition, processing, classification, and user interface design.

Many individual parts of a BCI system are typically first developed and evaluated on pre-existing datasets. However, there are only a few high quality publicly available datasets on which new systems, tools, and technologies can be evaluated and compared. For example, the publicly available BCI competition datasets (Sajda et al., 2003; Blankertz et al., 2004, 2006) provide an excellent set of resources for BCI researchers and have been widely used by numerous researchers to develop and evaluate new signal processing and classification methods (Arvaneh et al., 2013; Ghaemi et al., 2017; Lotte et al., 2018; Sakhavi et al., 2018; Zanini et al., 2018; Zhang et al., 2018). Yet, the relatively small size and number of such datasets introduce the risk of overfitting to methods developed and evaluated with these datasets. In other words, the reliability and reproducibility of BCI research is held back by a lack and sparsity of publicly available datasets.

This special issue provides a collection of descriptions of publicly available physiological datasets recorded during development, training, and evaluation of non-invasive BCI systems from BCI research labs around the world.

The collected datasets consist of signals recorded via a wide variety of modalities, including, but not limited to, electroencephalography (EEG), functional near infrared spectroscopy (fNIRS), electromyography (EMG), electrocardiography (ECG), galvanic skin response (GSR), skin temperature measures, respiration rates, and body movement data. Many datasets include multi-modal recordings with combinations of two or more of these signal modalities.

Data from a wide variety of different BCI paradigms are described. These include development of novel event-related potential (ERP) and steady state visual evoked potential (SSVEP) based BCIs for communication, motor imagery BCIs, affective BCIs, collaborative BCIs, and neurofeedback-based BCIs for nicotine addiction, as well as resting-state data.

Data on ERP-based BCIs are provided by several authors. For example, Delijorge et al. describe an EEG-based P300-based robotic hand control BCI; Simões et al. provide a large EEG-based P300-based BCI dataset; Li et al. implemented an ERP-based BCI for communication.

Motor control-based BCIs and associated datasets are also included in this collection. For example, Brandl and Blankertz provide an EEG dataset recorded during motor imagery while distractions were presented to simulate day-to-day events experienced outside the lab. Schwarz et al. made an attempt to decode reach and grasp actions from the EEG. Ortega et al. collected a multimodal dataset comprising EEG, fNIRS, EMG, and movement data recorded during a force grip task.

A wide range of other types of EEG-based BCIs are also presented. These include a dataset for a BCI based on covert attention shifts (Reichert et al.) and an affective BCI based on neurofeedback (Charles et al.), as well as two BCIs based on the rapid serial visual presentation paradigm (Zhang et al.; Zheng et al.). The collection also includes a BCI for treating nicotine addiction via neurofeedback (Bu et al.) and a dataset of SSVEP signals (Liu et al.).

A diverse range of paradigms were used in this collection of studies. For example, von Lühmann et al. present a resting state fNIRS dataset, while Parent et al. provide a multimodal dataset, comprising EEG, ECG, and respiration activity, recorded during a range of physical activities and induced stress. Finally, Albuquerque et al. offer a multimodal dataset, comprising EEG, ECG, and GSR, recorded during a mental workload paradigm.

We expect that the collected datasets will enable novel developments and applications of BCI technology, as well as extensive validation studies of current and future BCIs.

## Author Contributions

All authors co-wrote the editorial and edited the Research Topic.

## Funding

ML was supported by the Russian Science Foundation grant 21-75-30024.

## Conflict of Interest

DV is employed by Neurable Inc. The remaining authors declare that the research was conducted in the absence of any commercial or financial relationships that could be construed as a potential conflict of interest.

## Publisher's Note

All claims expressed in this article are solely those of the authors and do not necessarily represent those of their affiliated organizations, or those of the publisher, the editors and the reviewers. Any product that may be evaluated in this article, or claim that may be made by its manufacturer, is not guaranteed or endorsed by the publisher.
